# Sacrococcygeal teratoma

**DOI:** 10.4103/1817-1745.66682

**Published:** 2010

**Authors:** Arun Srivastava, Awadhesh K. Jaiswal, Kapil Jain, Sanjay Behari

**Affiliations:** Department of Neurosurgery, Sanjay Gandhi Postgraduate Institute of Medical Sciences, Lucknow, India

**Keywords:** Sacrococcygeal teratoma, radiological features, surgery

## Abstract

This neuroimage describes the clinicoradiological presentation of a Type II sacrococcygeal teratoma and summarizes its pathological features, its radiological presentation and its surgical management

Sacrococcygeal teratomas (SCT) are neoplasms composed of diverse tissues foreign to the anatomical site of origin (incidence: 1–2 per 40,000 deliveries). They originate from totipotent cells from Hansen’s node or primitive germ cells.[1]

A 6-day-old female child, following normal delivery, was born with a sacrococcygeal lump with no apparent neurological deficit. A sacrococcygeal swelling (sized 8 cm X 9 cm) was present with a variable solid to cystic consistency, transilluminant in certain areas [Figure 1]. Magnetic resonance imaging revealed a multiloculated swelling between the sacrococcygeal spine and the rectum [Figure 2].

**Figure 1:**
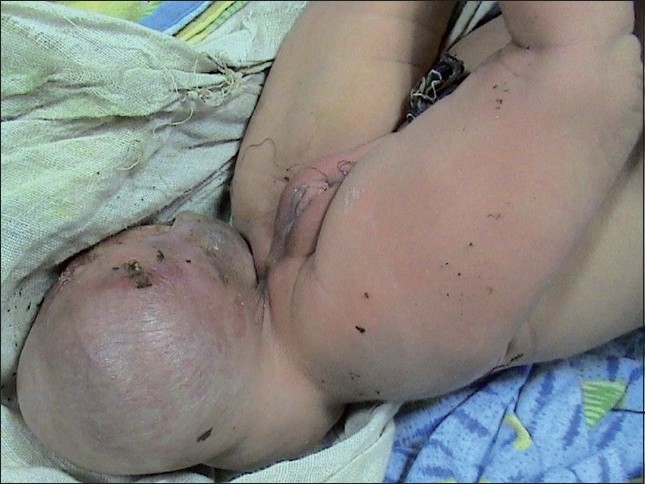
A sacrococcygeal swelling (sized 8 cm × 9 cm) was present with a variable solid to cystic consistency, transilluminant in certain areas

**Figure 2:**
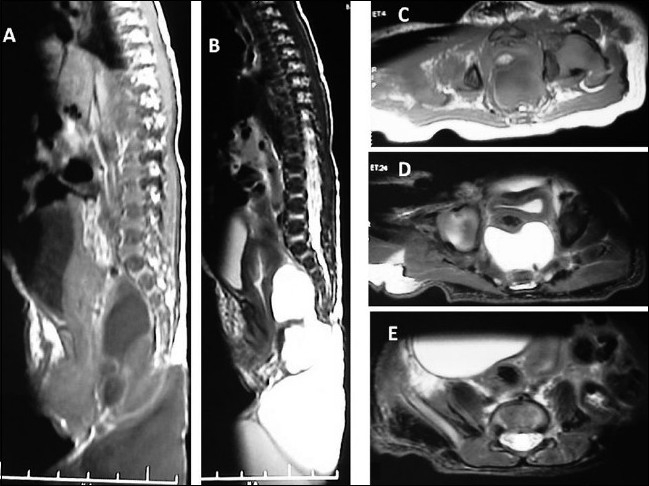
Sagittal T1 (A), T2 (B) and axial (C–E) magnetic resonance imaging revealed a multiloculated dumbell (both internal and external type) swelling between the sacrococcygeal spine and anorectal canal

SCT are derived from all three germinal layers and contain neural elements, squamous and intestinal epithelium, skin appendages, teeth and, at times, calcium. Their inheritance may be sporadic but, occasionally, autosomal dominant.[1,2] They are more common in girls (4:1) but are more often malignant in boys. Fifteen percent have associated congenital anomalies like imperforate anus, sacral bone defects, duplication of uterus or vagina, spina bifida and meningomyelocele (scimitar sacrum, anorectal malformation and presacral mass forming Currarino’s triad).[1–3]

The American Association of Pediatric Surgery has classified them into four types: (1) 47%, exophytic and external; (2) 34%, dumbbell shaped, with equal internal/external components (present patient); (3) 9%, primarily located within the abdomen/pelvis; (4) 10%, entirely internal, no external components. In a viable foetus, caesarean section delivery is recommended if SCT measures >5 cm to prevent dystocia or rupture of SCT. Early excision of the tumor in continuity with the coccyx should be achieved as failure to remove the coccyx results in 30–40% risk of local recurrence. Excess or damaged skin should be removed to allow a cosmetic reconstruction. The perianal sphincteric muscles are sutured to the presacral space.[1,2] In type 2/3 SCT, a combined abdominoperineal approach may be required. Malignant tumors require surgical excision, chemotherapy and radiation. Intrauterine surgery by interrupting the feeding vessel has been suggested when the fetus develops hydrops prior to viability. The role of open intrauterine surgery, however, is limited because it carries high morbidity to the mother and the fetus. Radiofrequency ablation has shown promising results in the treatment of fetal SCT.[1]

Risk of malignancy depends on the time of diagnosis (7–10% at <2 months, 37% at 1 year and 50% at 2 years). In benign tumors, disease-free survival is >90%, whereas malignant tumors have significant mortality. Hence, frequent follow-up with serum α-fetoprotein level measurement and surveillance by imaging is recommended.
